# Oncologic outcomes of microscopic tumor cut through in locally advanced oral squamous cell carcinoma^☆^^☆☆^^★^

**DOI:** 10.1016/j.bjorl.2025.101624

**Published:** 2025-05-14

**Authors:** Guilherme Reimann Agne, Hugo Fontan Kohler, Thiago Celestino Chulam, Clóvis Antônio Lopes Pinto, José Guilherme Vartanian, Luiz Paulo Kowalski

**Affiliations:** a Cirurgia de Cabeça e Pescoço no Grupo PESCOP, Balneário Camboriú, SC, Brazil; b A.C. Camargo Cancer Center, Departamento de Cirurgia de Cabeça e Pescoço e Otorrinolaringologia, São Paulo, SP, Brazil; c A.C. Camargo Cancer Center, Departamento de Patologia, São Paulo, SP, Brazil

**Keywords:** Mouth neoplasm, Frozen sections, Margins of excision

## Abstract

**Objective:**

This study aims to determine the impact of MTCT on local recurrence and disease-specific survival in patients with locally advanced T3-T4 OCSCC and compare it with other clinicopathological variables.

**Methods:**

A retrospective database analysis of patients diagnosed with locally advanced T3-T4 OCSCC surgically treated and submitted to intraoperative frozen section guiding the margin status. Survival was analyzed using the Kaplan-Meier estimator followed by the Cox model for multivariate analysis.

**Results:**

We analyzed 475 patients who met inclusion criteria: MTCT occurred in 29 patients (6.11%) and local recurrence was observed in 131 patients (27.6%). MTCT had an impact on univariate (HR = 2.205; 95% CI 1.243–3.914; *p* = 0.007) and multivariate (HR = 1.851; 95% CI 1.285–2.666; *p* = 0.001) analyses. Similar results were found for disease-specific survival: univariate (HZ = 1.669; 95% CI 1.056–2.635; *p* = 0.028) and multivariate (HZ = 1.307; 95% CI 0.816–2.092; *p* = 0.265) analyses. A total of 231 patients (48.6%) had died of cancer by the end of follow-up. The best predictor for compromised frozen sections was tumor depth of invasion.

**Conclusion:**

Even after negative final margins, MTCT is an important factor associated with poorer outcome, and treatment intensification should be considered in these patients.

**Level of evidence:**

Level III.

## Highlights

• MTCT had an impact on local recurrence on univariate (HR = 2.205;) and multivariate (HR = 1.851) analyses.
• Disease-specific survival was also affected on univariate (HZ = 1.669).
• The best predictor for compromised frozen sections was tumor depth of invasion.
• MTCT is an important factor associated with poorer outcome, and treatment intensification should be considered.

## Introduction

The gold standard treatment of Oral Cavity Squamous Cell Carcinoma (OCSCC) is surgical resection with free margins.^1^, ^2^, ^3^ Many clinicopathologic variables indicate adjuvant treatment to improve oncologic control. Radiotherapy is recommended for T3-T4 lesions, N2 or N3 nodal disease, perineural invasion, or vascular invasion,^1^, ^3^, ^4^, ^5^ whereas chemoradiotherapy is indicated for positive margins and extranodal extension.^6^, ^7^

The presence of positive final margins establishes worse outcomes, but the concept of clear and close margins is not a consensus in the literature.^8^, ^9^, ^10^ Most cancer centers use the following pattern of free margins: 1 cm healthy tissue around the tumor on macroscopic evaluation and 0.5 cm on microscopic evaluation.^11^ Intraoperative frozen section is widely used in Head and Neck Surgery to ensure a free margin status in final result.^12^, ^13^ Nevertheless, Microscopic Tumor Cut-Through (MTCT) occurs when an intraoperative frozen section margin is positive and is cleared with further resection to negative, and this event could define a worse outcome.^14^ Bulbul et al.^15^ conducted a meta-analysis of early stage OCSCC and showed that patients with MTCT have a better prognostic than those with positive margins and a worse prognostic than those with negative margins at first resection.^15^ Other studies that include all stages of oral cancer have reported similar results.^16^, ^17^ The real prognostic impact and therapeutic implication of MTCT is still under debate.^17^

This study aims to determine the impact of MTCT on local recurrence and disease-specific survival in patients with locally advanced OCSCC and compare it with other clinicopathological variables.

## Methods

A retrospective analysis of all patients diagnosed with cT3-cT4 OCSCC surgically treated in the aforementioned institution between 1985 and 2015 and submitted to intraoperative frozen section biopsy of surgical margins. The study was approved by the A.C. Camargo Cancer Center research board under nº 2532/18. We excluded patients with positive final margins in the last frozen section or final pathologic examination prior to head and neck cancer treatment. Demographic, clinical, surgical and pathological data of all patients were included in a database.

Statistical analysis was performed using the Stata 15.1 software for MacOS (Stata Corp, College Station, TX – USA). Categorical variables are described as frequency, and continuous variables as mean and standard deviation. Survival was analyzed using the Kaplan-Meier estimator followed by the Cox model for multivariate analysis. The outcomes of interest were local recurrence and disease-specific survival. All tests were two-tailed and statistical significance was considered when the p-value was ≤0.05.

## Results

We analyzed 475 consecutive patients: 399 men (84%) and 76 women (16%) aged 20–94 years (mean = 56.2 years, SD ± 11.6). The primary tumor was staged as pT3 in 258 patients (54.8%) and pT4a in 213 patients (45.2%). Neck dissection was performed in 468 patients 98.5%). Distribution of pathological neck stage/number of patients (%) was as follows: cNx/7 (1.5%), cN0/171 (36.0%), cN1/97 (20.4%), cN2a/6 (1.3%), cN2b/22 (4.6%), cN2c/70 (14.7%), and cN3/102 (21.5%). Bone involvement was diagnosed in 72 patients (19.7%), vascular invasion in 234 (50.6%) and neural invasion in 220 (48.6%). Tumor Depth Of Invasion (DOI) ranged from 10 to 60 mm (median of 14.48 mm).

MTCT occurred in 29 patients (6.1%). Postoperative radiotherapy was used in 312 patients (65.7%). Radiotherapy alone was used in 213 patients (44.8%) and 99 patients (20.8%) underwent concomitant chemoradiation. Radiotherapy alone or associated with chemotherapy were equivalent, and there was no statistically significant difference between them (HR = 1.67; 95% CI 0.891–2.382; *p* = 0.463). Follow-up time ranged from 0.3 to 239.6 months (mean = 46.4 months, SD ± 20.7). Local recurrence was diagnosed in 131 patients (27.6%). Univariate analysis showed pT stage, lymphatic invasion, and MTCT as significant factors associated with local recurrence (Table 1). Tumor DOI, perineural invasion, and bone involvement were not significant. In multivariate analysis, vascular invasion and MTCT remained as significant variables (Table 2).

**Table 1:** Univariate analysis of local recurrence.

Variable	Values	HR	95% CI	p-value
pT stage	pT3	1		
	pT4a	1.425	1.008‒2.105	0.045
Vascular Invasion	No	1		
	Yes	1.927	1.342‒2.766	<0.001
MTCT	No	1		
	Yes	2.205	1.243‒3.914	0.007

HR, Hazard Ratio, CI, Confidence Interval; MTCT, Microscopic Tumor Cut-Through.

**Table 2:** Multivariate analysis of local recurrence.

Variable	Values	HR	95% CI	p-value
pT stage	pT3	1		
	pT4a	1.327	0.930‒1.893	0.119
Vascular Invasion	No	1		
	Yes	2.054	1.152‒3.663	0.015
MTCT	No	1		
	Yes	1.8551	1.285‒2.666	0.001

HR, Hazard Ratio, CI, Confidence Interval; MTCT, Microscopic Tumor Cut-Through.

At the end of follow-up, 121 patients (25.5%) were alive with no evidence of disease, 31 patients had complication-related deaths (6.5%), 80 patients had died of other causes (16.8%), 12 patients (2.5%) completed the 1-year follow-up, and 231 patients (48.6%) had died of cancer. Univariate survival analysis with disease-specific survival as the outcome of interest showed pT and pN stages, perineural and vascular invasions, and MTCT as significant variables (Table 3). MTCT was not significant in the multivariate analysis (Table 4).

**Table 3:** Univariate analysis of disease-specific survival.

Variable	Values	HR	95% CI	p-value
pT stage	pT3	1		
	pT4a	1.600	1.250‒2.048	<0.001
pN stage	pNx-0			
	pN1	2.077	1.349‒3.201	0.001
	pN2	4.036	2.445‒6.663	<0.001
	pN3	9.455	2.292‒19.001	0.002
Perineural invasion	No	1		
	Yes	1.633	1.269‒2.101	<0.001
Vascular Invasion	No	1		
	Yes	1.570	1.222‒2.019	<0.001
MTCT	No	1		
	Yes	1.669	1.056‒2.635	0.028

HR, Hazard Ratio; CI, Confidence Interval; MTCT, Microscopic Tumor Cut-Through.

**Table 4:** Multivariate analysis of disease-specific survival.

Variable	Values	HR	95% CI	p-value
pT stage	pT3	1		
	pT4a	1.449	1.120‒1.874	0.005
pN stage	pNx-0	1		
	pN1	1.844	1.187‒2.250	0.010
	pN2	2.659	1.561‒4.529	<0.001
	pN3	9.525	3.131‒14.532	<0.001
Perineural invasion	No	1		
	Yes	1.386	1.070‒1.796	0.013
Vascular Invasion	No	1		
	Yes	1.351	1.039‒1.757	0.025
MTCT	No	1		
	Yes	1.307	0.816‒2.092	0.265

HR, Hazard Ratio; CI, Confidence Interval; MTCT, Microscopic Tumor Cut-Through.

**Fig. 1:**
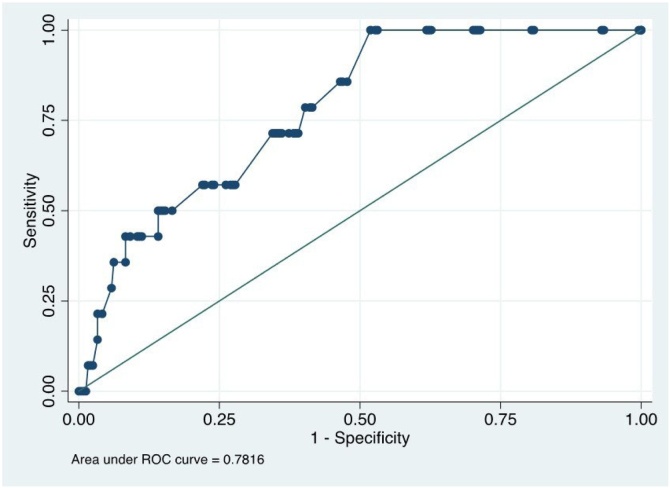
ROC (Receiver Operator Characteristic) curve for compromised surgical margins according to tumor DOI.

The best predictor for positive frozen section is tumor DOI measured as a continuous variable (Fig. 1).

## Discussion

The values found in our sample for Disease-Specific Survival (DSS) and Local Recurrence (LR) are similar to those of other studies conducted with similar populations.^15^, ^16^, ^17^ MTCT occurred in 6.1% of patients, and its impact on outcome was evident. Both univariate and multivariate analyses presented statistical significance for the presence of a positive frozen section in LR, demonstrating that MTCT is an independent prognostic factor. DSS was statistically valid only in the univariate analysis. Patel et al.^17^ conducted a similar cohort study with T1-T4 OCSCC patients and found that MTCT occurred in 9.6% of them.^17^ In this same study, the outcome analysis was similar to ours, and the lack of significance in the multivariate analysis for DSS is explained because most cases of MTCT also presented lymph node disease and other compromising factors.^17^

Relationship between MTCT and worse prognosis has been reported in the literature since 1986.^14^ Scholl et al.^14^ reported worse oncological outcome in these patients and predicted worse biological behavior of tumor as a causal factor.^14^ Currently, the peculiar local dissemination pattern of OCSCC is considered the most significant factor responsible for treatment local failures.^17^, ^18^

When OCSCC grows in depth, it reaches the musculature, neurovascular bundles, and glandular and areolar tissues. These structures could act as avenues that facilitate locoregional progression.^18^ Thereby, in the present study, we observed that the main factor related to MTCT is tumor DOI, as shown by the ROC curve in Fig. 1. This reinforces the hypothesis that MTCT may not be a true surgical tactical error, but that worse outcome results from the presence of a tumor with greater DOI and a more complex invasion pattern.^17^, ^18^, ^19^

DOI has been gaining emphasis in the literature.^19^, ^20^ The last TNM staging system (8th edition) included DOI at the “T” status, thus upscaling oral cancer clinical stage.^20^, ^21^ The importance of tumor DOI in contrast to a two-dimensional view of the tumor has motivated some authors to propose the concept of compartmental resection for tongue and floor of mouth cancer.^18^, ^19^, ^22^ This technique proposes continuous removal of tumor within the musculature of the tongue and the entire area at risk of vascular invasion, perineural invasion, and lymphatic dissemination between primary tumor and neck lymph nodes.^18^, ^19^

Frozen section analysis is always a reason for discussion, and this technique is far from being standardized.^11^, ^23^, ^24^, ^25^ An important fact that should be considered is whether resection after a positive frozen section is made in the wrong spot.^12^ Sampling is also controversial in many cancer centers, although most studies have reported benefits in sampling the surgical specimen instead of the patient.^12^ We have standardized frozen section sampling of surgical specimen in our hospital, and the importance of the interaction between the surgical and pathology teams in the analysis of intraoperative margin must be highlighted.

Currently, radiotherapy is not indicated after MTCT1. Patel et al.^17^ considered, based on subgroup analysis, that in the absence of nodal involvement, MTCT do not present sufficient outcome impact to indicate adjuvant therapy in T1-T2 OCSCC.^17^ In this study, 65.7% of T3-T4 OCSCC patients received adjuvant radiotherapy. The low proportion of patients receiving adjuvant radiotherapy is probably due to the retrospective and old nature of our data, since standardization of adjuvant treatment indication is recent.^26^, ^27^ Nevertheless, no patients received chemoradiotherapy, and this combined adjuvant therapy should be considered in advanced OCSCC cases with MTCT.^6^, ^7^

## Conclusion

The relation between DOI and MTCT indicates tumors with a more complex infiltration pattern, and a more extensive resection should be considered. Even after negative final margins, in this large retrospective series, MTCT is an important predictive factor of poorer OCSCC outcomes. In the absence of a prospective clinical trial, the decision for more aggressive treatment should be considered in an individual basis. In this scenario, we suggest that decision about using adjuvant treatment be discussed in a tumor board to evaluate MTCT together with other prognostic indicators.

## Financial support

None.

## Declaration of competing interest

The authors declare no conflicts of interest.
